# Ultra‐short‐term versus short‐term measures of heart rate variability in specialist police units: A pilot reliability study

**DOI:** 10.14814/phy2.70182

**Published:** 2025-01-16

**Authors:** Colin D. Tomes, Elisa F. D. Canetti, Ben Schram, Robin Orr

**Affiliations:** ^1^ Faculty of Health Science and Medicine Bond University Robina Queensland Australia; ^2^ Tactical Research Unit Bond University Robina Queensland Australia

**Keywords:** biosignals, conditioning, physiological monitoring, police, resilience, stress

## Abstract

Police officers are exposed to high levels of stress. Serving on Special Weapons and Tactics (SWAT) teams is a highly demanding duty that may further increase levels of stress in police personnel. This stress may accumulate, thereby increasing allostatic load. As such, holistic stress measures may be valuable for quantifying multifactorial stress accumulation in SWAT personnel. Heart rate variability (HRV) is one field‐deployable measure that may be suitable in this context. However, with logistical challenges present in this population, determining if 30 s; rather than more the typical 5‐min ECG data collection, provides sufficient reliability may be beneficial for reducing the logistical barrier to adoption of HRV monitoring in SWAT personnel. This study compared 30‐s to 5‐min HRV analyses of ECG data obtained from 15 male SWAT personnel. Findings demonstrated good (ICC >0.8) reliability only in the VLF, HF, SD1, and SD2 HRV domains. The VLF and SD2 measures may be erroneous, as 5‐min may still insufficiently characterize these measures. However, this study confirms the robust quality of nonlinear HRV analysis, as the SD1 value demonstrated the highest ICC reported here (0.902). Therefore, while 5‐min ECGs may still preferable, the 30‐s measure may still be viable for organizations considering HRV assessment.

## 1 INTRODUCTION

Law enforcement professionals are exposed to high levels of physical, mental, and emotional stress (Gilmartin, 2002). Physical stressors may present as challenging and demanding tasks of a physical nature, including pursuing offenders on foot, lifting and carrying heavy loads, evacuating casualties, subduing offenders, and performing first response during a natural disaster (Lockie et al., 2018). Duties and tasks that are mentally and emotionally challenging may also be commonly encountered, with examples including performing emergency life‐saving measures, intervening during violent altercations, and resolving interpersonal conflicts (Orr, Hinton, et al., 2020). Not only are these demanding events often spontaneous occurrences in unpredictable environments, but recovery opportunities between incidents may also be limited. Further, the daily duties of a police officer are often compounded by the carriage of heavy external loads of around 10 kg (Baran et al., 2018; Ramstrand et al., 2016), the imposition of environmental stress (such as extreme heat and humidity or cold), and exposure to social stressors associated with critical incident response (Carbone et al., 2014).

In addition to the external demands imposed by responding to emergencies, personnel are also subject to internal organizational demands. Such demands include irregular work hours (rotating shift schedules, overtime due to understaffing, etc.), prolonged shifts (such as remaining extended on duty during an emergency), and collateral duties (such as work in search and rescue, crowd control, and mounted police) (Irving et al., 2019; Maupin et al., 2018). In the United States, serving on Special Weapons and Tactics (SWAT) teams serve as an example of a highly demanding collateral duty. The selection process for these units is generally physically challenging and technically demanding (Sax van der Weyden et al., 2021). Candidates are expected to demonstrate proficiency in a range of psychomotor skills, and the ability to learn and adapt to change quickly. These characteristics are assessed under extreme competitive pressure (Irving et al., 2019; Koepp, 2000). Furthermore, even once accepted onto a team, personnel must devote additional time to training, accept additional on‐duty hours, and respond to the most high‐risk scenarios within the region served by the organization (Green, 2001; Irving et al., 2019). As such, the health and wellness of these personnel is of interest not only for the team and its constituent members, but also for the organization and wider communities in which they serve (Green, 2001; Irving et al., 2019).

Given the multitude of stressors and pressures placed on police officers, and particularly officers serving in SWAT teams, the risk of allostatic load may be highly relevant to the overall health and wellbeing of members. Allostatic load describes insufficient or overactive response following prolonged exposure beyond tolerable levels of stress (Mancia et al., 2007; McEwen & Wingfield, 2003). As initially put forth by Bruce McEwen et al., allostatic load accumulation describes increased vulnerability to physiological maladaptation as an individual is exposed to stress levels that exceed their capacity (McEwen & Stellar, 1993; McEwen & Wingfield, 2003). While the relationship between allostasis and work in policing is still emerging (Corrigan et al., 2021), assessing for diminished dynamic and adaptive regulatory responses in this population may be key for early detection and intervention. This is because one of the chief hallmarks of allostatic load is persistent overactivity of stress responses associated with immediate survival of threats to life. Therefore, detecting the onset of allostatic load may be of critical value, particularly in those individuals who are not only at a potentially increased risk of allostatic load, but who also carry out tasks essential for the safety of the communities they serve.

While allostatic load is a complex construct to estimate, research in the military setting has pointed to heart rate variability (HRV) as a plausible in situ measure (Macartney et al., 2022). HRV is detected by computing the dynamic oscillations of cardiac activity, either R‐R intervals (RRI) from an electrocardiographs (ECG) or normal‐normal intervals derived from a pulse plethysmograph (PPG). HRV results from complex interactions of intrinsic cardioregulatory factors (i.e., cardiac conduction system, ganglionated fat pads, and natriuretic peptides) as well as the sympathetic (SNS) and parasympathetic (PNS) nervous systems, amongst other extrinsic influences (Shaffer & Ginsberg, 2017; Thayer et al., 2012). HRV is therefore utilized as a measure for quantifying stress in tactical populations, such as the military, fire and rescue, and law enforcement (Andrew et al., 2017; Gamble et al., 2018; Rodrigues et al., 2018). Indeed, HRV has shown promise as a viable field measurement for determining maladaptive stress responses in a variety of field environments (Andrew et al., 2012; Shin et al., 2016). However, a multiplicity of measurements and approaches to HRV analysis have been reported in the literature (Sammito et al., 2015). Mostly typically, HRV research designates data collection epochs as long term (LT, >10 min to 24 h), short term (ST, 1–2 min to 5 min) and ultra‐short term (UST, 10–30 s) (Shaffer & Ginsberg, 2017). While 24‐h ECG recordings are generally considered the gold standard measurement, shorter recordings may still be sufficiently robust and are generally more practically achieved in a tactical setting (Stephenson et al., 2021). Intervals as short as 10–30 s have been described in the literature and have demonstrated utility but may come at the cost of some sacrificed precision in that shorter epochs may not consistently describe slower oscillations driving some HRV patterns (Burma et al., 2021; Shaffer et al., 2020). However, given that longer data collections periods may not be practical or even possible in many tactical units, exploring the utility of very brief data collection windows may be valuable. Therefore, the objective of this study was to determine the reliability of UST HRV measurements as compared to ST measurements in a cohort of SWAT personnel.

## 2 MATERIALS AND METHODS

This study was a prospective investigation of 15 male specialist police officers with an average of 5.2 ± 4.4 years of experience as SWAT members. All team members present for a regularly scheduled training exercise were eligible for inclusion; this included females, but none were present at the time of data collection. Personnel prescribed cardioactive medications, medications for a renal, respiratory, or neurological condition, or who disclosed an injury or illness were excluded. Personnel were not to utilize tobacco products for the duration of the exercise. All personnel were recruited, consented, and followed for data collection on a single day of training. All data were collected during a regularly scheduled training event at the team's training grounds. Some personnel (*n* = 10) were scheduled for a shift prior to the qualification event (described below), and others were off duty the night before (*n* = 6). Which personnel were on duty, and which were off duty was not decided by or known to the research team until after the qualification event was complete. No further descriptive data were available as per a privacy agreement with the team, which has occurred in other published studies investigating similar populations (Orr, Robinson, et al., 2020).

All personnel provided their informed written consent, and the unit commander provided permission for publication of this work. The research protocol was approved by the Messiah University Institutional Review Board (2019–022) and the Bond University Human Research Ethics Committee (2019–022 amendment 2). All procedures were conducted in accordance with the Declarations of Helsinki and its later amendments (Williams, 2008).

Three‐lead seated ECGs were obtained for 5 min prior to the start of training, between 8:30 and 9:15 am, and again immediately after the completion of yearly firearms qualification (9:15–10:15 am). Personnel were all require personal protective equipment for all ECG measures. To compare this typical short‐term window against a comparable ultra‐short‐term window, HRV values derived from the initial 30 s of each ECG trace were compared against values derived from the entire 5‐min trace. While data were being collected, personnel were asked to sit quietly, refrain from moving, and avoid using cell phones or talking. ECGs were obtained with an ADInstruments Powerlab (ADInstruments, Sydney, Australia) with leads placed on both wrists over the distal/lateral radius close to the where the radial artery is typically palpated. The final lead was placed on the left ankle, just above the medial malleolus. Leads were not placed on the chest so as to facilitate data collection without requiring participants to remove their personal protective equipment. LabChart (v7, ADInstruments, Sydney, Australia) software was used to capture and record all ECG data. The sampling frequency was 20KHz.

**FIGURE 1:**
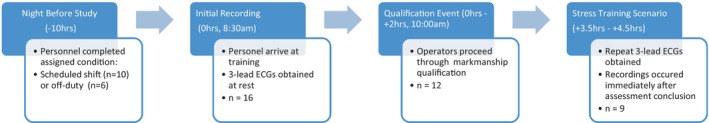
Sequence of participant duties and data acquisition.

The qualification event has been described in previous literature (Tomes et al., 2021) and consisted of a handgun (Glock 22 handgun firing 0.40 cal S&W ammunition) and rifle (M4 rifle firing 5.56 mm ammunition) shoot, with one attempt permitted per weapon. The handgun qualification was a static engagement of targets at 3, 5, 7, 10, 15, and 25 m. The rifle qualification consisted of target engagements at 50, 25, and 15 m with shots delivered while walking down to the final closest distance of 5 m. Personnel were required to wear all standard issue gear (boots, uniform, plate carrier vest, helmet) and accurately place shots on all targets at all distances. The assessment was pass/fail, with 100% accuracy required to pass; failure resulted in elimination from the team (but not the agency), and passing represented a significant yearly milestone for team personnel. All personnel present at the initial data collection period completed the qualification event within 2 h of the initial baseline recording (8:30–10:30 am). After completing the qualification and receiving notice of pass or failure, participants immediately reported for their second HRV measurement (9:15–10:15 am). After being provided a meal break, operators returned for a stress training scenario and reported for the final ECG assessment from 1:45 pm to 2:45 pm. The stress training scenario was formative in nature only, and personnel were not formally assessed in this phase. See Figure 1 for additional details on the timeline of events and data acquisition. Due to some personnel being called away for other police tasks, the largest sample size for reliability comparison was this baseline measurement period; some attrition is noted in the later analyses.

ECG data were processed with a LabChart v8 student license (ADInstruments, Sydney, Australia). Visual ECG examination was performed in combination with visual analysis of RRI plots to exclude outlier and ectopic beats. The boundaries of the RRI plots were determined by ECG complexity, a measurement of QRS complex quality provided by the LabChart software, and were set between 0.9 and 1.4. The other determining factors of the RRI plot was RRI time. Acceptable RRIs were established as those between 272 and 1600 ms. This is equivalent to an HR between 220 and 37.5 bpm (Sammito et al., 2015). Any RRI outside of this range was manually reviewed and included or excluded based on the visual features of the ECG.

HRV was defined across the following commonly reported methods: standard deviation of R‐to‐R intervals (SDRR), heart rate (HR), root‐mean square of successive differences (RMSSD), percentage of R‐to‐R intervals varying by at least 50 ms (pRR50), total spectral power (TP), very‐low frequency (VLF), low frequency (LF), and high frequency (HF). The spectral indices were calculated by fast fourier transform. Nonlinear Poincare plot standard deviation along the line perpendicular to the identity (SD1) and Poincare plot standard deviation along the line of identity (SD2) were also included. For statistical analysis of comparability (reliability and agreement between measures) between the 30 s (UST) and 5‐min (ST) measure, intraclass correlation coefficients (ICCs) and Bland–Altman plots were calculated for each HRV domain, along with HR in JASP (v0.18, JASP Project, University of Amsterdam, Amsterdam, The Netherlands). Interpretation of the ICCs were conducted in accordance with cited literature within JASP (Koo & Li, 2016); based on the 95% confidence interval of the ICC estimate, values less than 0.5, between 0.5 and 0.75, between 0.75 and 0.9, and greater than 0.90 are indicative of poor, moderate, good, and excellent reliability, respectively.

## 3 RESULTS

All personnel passed the qualification event following this initial data collection window. Descriptive values of each measure and time period are reported in Tables 1, 2, 3, 4, 5, 6. Signal quality was generally high, with excluded beats limited to 1.25 ± 1.24% in the baseline condition, 0.92 ± 1.31% in the post‐qualification condition, and 0.67 ± 1.32% in the post‐stress shoot condition. Results of the ICC analysis between 30 s and 5‐min values determined good or better reliability for the following HRV domains at baseline: SDRR, RMSSD, VLF, HF, SD1, and SD2 (Table 7). No other HRV domains demonstrated acceptable reliability values. Results of the ICC analysis for the post‐qualification data determined good or better reliability for pRR50% and for HR (Table 8). No HRV domains were acceptably reliable for the post‐stress event data, but HR was (Table 9).

**TABLE 1:** Initial UST values.

	SDRR (ms)	HR (bpm)	RMSSD (ms)	pRR50 (%)	TP (ms^2^)	VLF (ms^2^)	LF (ms^2^)	HF (ms^2^)	SD1 (ms)	SD2 (ms)
Mean	39.86	82.16	27.38	8.62	3012.89	604.20	1426.62	951.63	19.53	52.28
SD	20.97	24.58	20.61	12.00	7060.96	1483.67	3414.91	2089.36	14.78	26.97
Median	37.84	84.47	22.02	2.89	808.90	151.05	435.45	215.75	15.50	52.56
Min	15.15	5.17	5.86	0.00	91.69	3.79	34.66	10.11	4.16	20.60
Max	92.16	109.70	84.62	40.00	29190.00	6095.00	14090.40	8440.00	60.43	115.50
Range	77.01	104.54	78.77	40.00	29098.31	6091.21	14055.74	8429.89	56.27	94.90

*Note*: Range computed and reported as the difference between the maximum and minimum value for each series.

Abbreviations: HF, High frequency; HR, Heart rate; LF, Low frequency; Max, Maximum value for the series; Min, Minimum value for the series; RMSSD, Root‐mean square of successive differences; SD, Standard Deviation; SD1, Poincare plot standard deviation along the line perpendicular to the identity; SD2, Poincare plot standard deviation along the line of identity; SDRR, Standard deviation of R‐R Intervals; TP, Total Power; VLF, Very Low frequency.

**TABLE 2:** Initial ST values.

	SDRR (ms)	HR (bpm)	RMSSD (ms)	pRR50 (%)	TP (ms^2^)	VLF (ms^2^)	LF (ms^2^)	HF (ms^2^)	SD1 (ms)	SD2 (ms)
Mean	49.98	84.26	28.21	8.45	2632.66	1096.49	1014.44	495.97	19.98	69.30
SD	22.36	13.02	21.04	14.42	3107.32	1582.82	757.51	1111.79	14.89	28.62
Median	46.13	82.68	24.09	3.79	1644.00	614.20	793.80	139.30	17.12	66.05
Min	18.91	53.56	10.46	0.00	224.70	44.83	133.40	24.12	7.40	25.67
Max	117.20	107.30	99.27	58.89	13290.00	6561.00	3250.00	4596.00	70.29	150.00
Range	98.29	53.74	88.81	58.89	13065.30	6516.17	3116.60	4571.88	62.89	124.33

*Note*: Range computed and reported as the difference between the maximum and minimum value for each series.

Abbreviations: HF, High frequency; HR, Heart rate; LF, Low frequency; Max, Maximum value for the series; Min, Minimum value for the series; RMSSD, Root‐mean square of successive differences; SD, Standard Deviation; SD1, Poincare plot standard deviation along the line perpendicular to the identity; SD2, Poincare plot standard deviation along the line of identity; SDRR, Standard deviation of R‐R Intervals; TP, Total Power; VLF, Very Low frequency.

**TABLE 3:** Qualification Event UST values.

	SDRR (ms)	HR (bpm)	RMSSD (ms)	pRR50 (%)	TP (ms^2^)	VLF (ms^2^)	LF (ms^2^)	HF (ms^2^)	SD1 (ms)	SDRR (ms)
Mean	69.86	91.18	63.70	12.78	3681.94	318.49	1863.25	1364.66	45.54	84.57
SD	31.57	19.17	52.87	15.19	3891.80	495.05	2202.57	1904.53	37.81	33.87
Median	69.26	90.17	43.88	6.19	1665.00	176.70	949.45	536.55	31.15	83.77
Min	23.01	54.89	7.66	0.00	693.00	29.87	245.20	64.52	5.43	32.08
Max	118.60	128.40	158.20	48.00	11570.00	1869.00	7817.00	6199.00	113.00	149.50
Range	95.59	73.51	150.55	48.00	10877.00	1839.13	7571.80	6134.48	107.57	117.42

*Note*: Range computed and reported as the difference between the maximum and minimum value for each series.

Abbreviations: HF, High frequency; HR, Heart rate; LF, Low frequency; Max, Maximum value for the series; Min, Minimum value for the series; RMSSD, Root‐mean square of successive differences; SD, Standard Deviation; SD1, Poincare plot standard deviation along the line perpendicular to the identity; SD2, Poincare plot standard deviation along the line of identity; SDRR, Standard deviation of R‐R Intervals; TP, Total Power; VLF, Very Low frequency.

**TABLE 4:** Qualification Event ST values.

	SDRR (ms)	HR (bpm)	RMSSD (ms)	pRR50 (%)	TP (ms^2^)	VLF (ms^2^)	LF (ms^2^)	HF (ms^2^)	SD1 (ms)	SDRR (ms)
Mean	64.53	85.57	50.87	10.20	3612.44	1196.97	1119.39	1067.65	36.01	82.73
SD	33.84	15.75	40.39	16.94	4715.80	2212.27	946.05	1595.63	28.60	40.92
Median	55.38	85.69	40.02	2.31	1601.00	415.70	806.90	351.95	28.33	68.71
Min	29.33	53.85	10.22	0.19	666.70	127.00	261.60	68.18	7.23	40.34
Max	144.20	109.30	147.60	59.60	16250.00	8090.00	3000.00	4717.00	104.50	175.10
Range	114.87	55.45	137.38	59.41	15583.30	7963.00	2738.40	4648.82	97.27	134.76

*Note*: Range computed and reported as the difference between the maximum and minimum value for each series.

Abbreviations: HF, High frequency; HR, Heart rate; LF, Low frequency; Max, Maximum value for the series; Min, Minimum value for the series; RMSSD, Root‐mean square of successive differences; SD, Standard Deviation; SD1, Poincare plot standard deviation along the line perpendicular to the identity; SD2, Poincare plot standard deviation along the line of identity; SDRR, Standard deviation of R‐R Intervals; TP, Total Power; VLF, Very Low frequency.

**TABLE 5:** Stress Training Scenario UST values.

	SDRR (ms)	HR (bpm)	RMSSD (ms)	pRR50 (%)	TP (ms^2^)	VLF (ms^2^)	LF (ms^2^)	HF (ms^2^)	SD1 (ms)	SDRR (ms)
Mean	45.85	113.02	46.46	3.23	6813.85	615.21	1461.38	4068.74	33.22	52.59
SD	30.85	23.39	52.34	2.96	16867.36	1232.65	3056.20	10900.42	37.56	29.47
Median	42.74	109.50	18.76	2.17	910.90	19.80	150.50	188.00	13.31	58.97
Min	12.06	73.38	3.94	0.00	84.95	2.15	36.62	11.24	2.79	16.83
Max	103.40	148.70	146.80	7.14	51650.00	3536.00	9503.00	33080.00	105.70	101.00
Range	91.34	75.32	142.86	7.14	51565.05	3533.86	9466.38	33068.76	102.91	84.17

*Note*: Range computed and reported as the difference between the maximum and minimum value for each series.

Abbreviations: HF, High frequency; HR, Heart rate; LF, Low frequency; Max, Maximum value for the series; Min, Minimum value for the series; RMSSD, Root‐mean square of successive differences; SD, Standard Deviation; SD1, Poincare plot standard deviation along the line perpendicular to the identity; SD2, Poincare plot standard deviation along the line of identity; SDRR, Standard deviation of R‐R Intervals; TP, Total Power; VLF, Very Low frequency.

**TABLE 6:** Stress Training Scenario ST values.

	SDRR (ms)	HR (bpm)	RMSSD (ms)	pRR50 (%)	TP (ms^2^)	VLF (ms^2^)	LF (ms^2^)	HF (ms^2^)	SD1 (ms)	SDRR (ms)
Mean	39.12	104.70	20.72	2.34	1807.66	1424.75	432.13	73.72	14.66	52.76
SD	23.96	19.58	13.66	5.32	3409.85	3189.57	597.62	93.30	9.67	33.55
Median	33.13	105.30	17.68	0.56	344.50	199.00	151.10	33.33	12.51	42.04
Min	20.90	65.59	5.99	0.00	162.10	47.93	33.81	7.00	4.24	29.02
Max	98.18	129.20	37.30	16.47	10440.00	9785.00	1530.00	298.70	26.41	136.70
Range	77.28	63.61	31.31	16.47	10277.90	9737.07	1496.19	291.71	22.17	107.68

*Note*: Range computed and reported as the difference between the maximum and minimum value for each series.

Abbreviations: HF, High frequency; HR, Heart rate; LF, Low frequency; Max, Maximum value for the series; Min, Minimum value for the series; RMSSD, Root‐mean square of successive differences; SD, Standard Deviation; SD1, Poincare plot standard deviation along the line perpendicular to the identity; SD2, Poincare plot standard deviation along the line of identity; SDRR, Standard deviation of R‐R Intervals; TP, Total Power; VLF, Very Low frequency.

**TABLE 7:** Initial recording reliability analysis results.

HRV measure	Point estimate	Lower bound	Upper bound
SDRR (ms)	0.894^a^	0.724	0.962
HR (bpm)	0.357	−0.151	0.716
RMSSD (ms)	0.887^a^	0.708	0.959
pRR50 (%)	0.650	0.244	0.862
TP (ms^2^)	0.711	0.348	0.889
VLF (ms^2^)	0.945^b^	0.850	0.980
LF (ms^2^)	0.139	−0.368	0.582
HF (ms^2^)	0.813^a^	0.544	0.930
SD1 (ms)	0.885^a^	0.710	0.958
SD2 (ms)	0.881^a^	0.694	0.957

Abbreviations: HF, High frequency; HR, Heart rate; LF, Low frequency; RMSSD, Root‐mean square of successive differences; SD1, Poincare plot standard deviation along the line perpendicular to the identity; SD2, Poincare plot standard deviation along the line of identity; SDRR, Standard deviation of R‐R Intervals; TP, Total Power; VLF, Very Low frequency.

^a^ Indicates good reliability (Koo & Li, 2016).

^b^ Indicates excellent reliability (Koo & Li, 2016).

**TABLE 8:** Qualification event reliability analysis results.

HRV measure	Point estimate	Lower bound	Upper bound
SDRR (ms)	0.519	−0.047	0.833
HR (bpm)	0.944^a^	0.818	0.984
RMSSD (ms)	0.698	0.236	0.903
pRR50 (%)	0.923^a^	0.757	0.977
TP (ms^2^)	>0.001	−0.553	0.553
VLF (ms^2^)	0.000	−0.553	0.553
LF (ms^2^)	0.249	−0.353	0.705
HF (ms^2^)	>0.001	−0.553	0.553
SD1 (ms)	0.697	0.234	0.902
SD2 (ms)	0.441	−0.148	0.799

Abbreviations: HF, High frequency; HR, Heart rate; LF, Low frequency; RMSSD, Root‐mean square of successive differences; SD1, Poincare plot standard deviation along the line perpendicular to the identity; SD2, Poincare plot standard deviation along the line of identity; SDRR, Standard deviation of R‐R Intervals; TP, Total Power; VLF, Very Low frequency.

^a^ Indicates excellent reliability (Koo & Li, 2016).

**TABLE 9:** Stress training scenario reliability analysis results.

HRV measure	Point estimate	Lower bound	Upper bound
SDRR (ms)	0.436	−0.187	0.809
HR (bpm)	0.952^a^	0.832	0.987
RMSSD (ms)	0.360	−0.273	0.775
pRR50 (%)	0.402	−0.226	0.794
TP (ms^2^)	>0.001	−0.576	0.576
VLF (ms^2^)	0.238	−0.392	0.716
LF (ms^2^)	>0.001	−0.576	0.576
HF (ms^2^)	0.000	−0.576	0.576
SD1 (ms)	0.355	−0.278	0.773
SD2 (ms)	0.595	0.028	0.872

Abbreviations: HF, High frequency; HR, Heart rate; LF, Low frequency; RMSSD, Root‐mean square of successive differences; SD1, Poincare plot standard deviation along the line perpendicular to the identity; SD2, Poincare plot standard deviation along the line of identity; SDRR, Standard deviation of R‐R Intervals; TP, Total Power; VLF, Very Low frequency.

^a^ Indicates excellent reliability (Koo & Li, 2016).

### 3.1 Results of the Bland–Altman plots are found below

Bland–Altman plot analysis (Figures 2, 3, 4, 5, 6, 7, 8, 9, 10, 11) revealed measurements outside of the mean difference 95% confidence interval zone for all HRV measures at all time points. Specific values of the mean measurement differences and 95% CI bounds can be found in Table 10.

**FIGURE 2:**
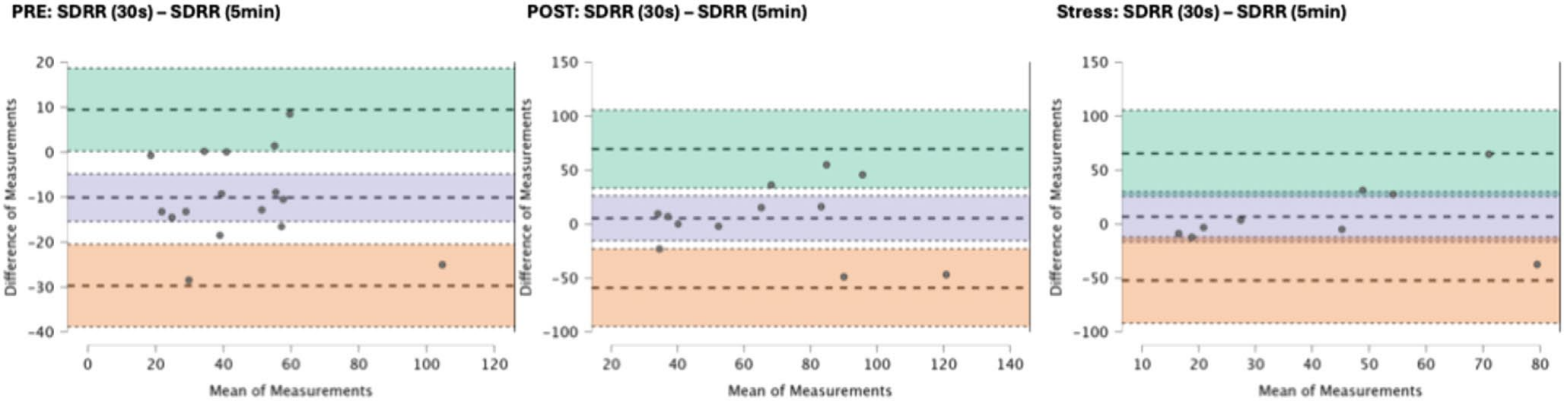
Bland–Altman plot of SDRR by timepoint. The purple region corresponds to the mean difference and 95% CI. The green region corresponds to the mean difference +1.96SD and 95% CI. The orange region corresponds to the mean difference −1.96SD and 95% CI.

**FIGURE 3:**
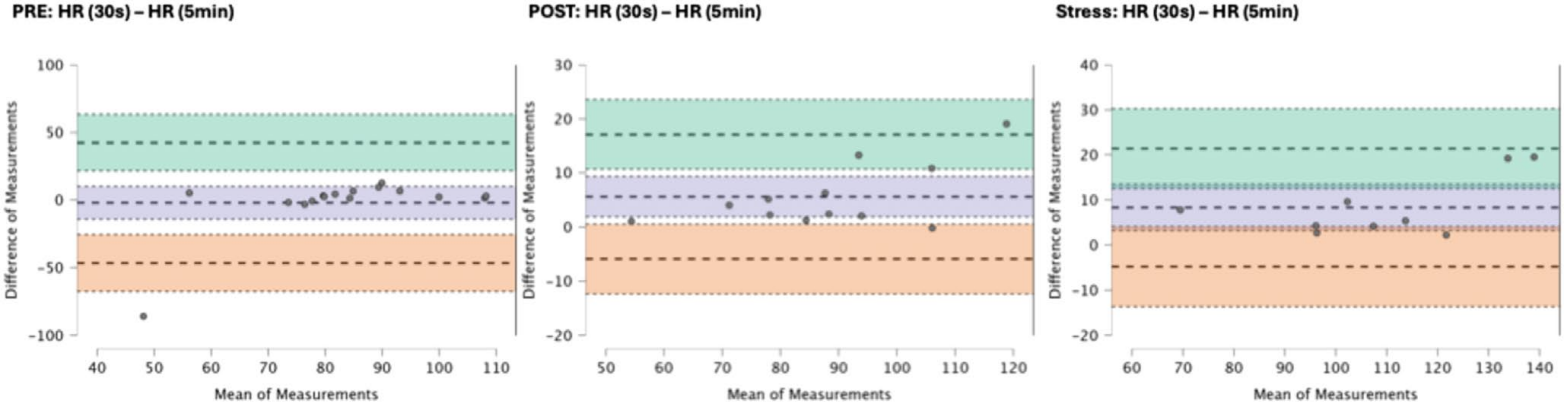
Bland–Altman plot of HR by timepoint. The purple region corresponds to the mean difference and 95% CI. The green region corresponds to the mean difference +1.96SD and 95% CI. The orange region corresponds to the mean difference −1.96SD and 95% CI.

**FIGURE 4:**
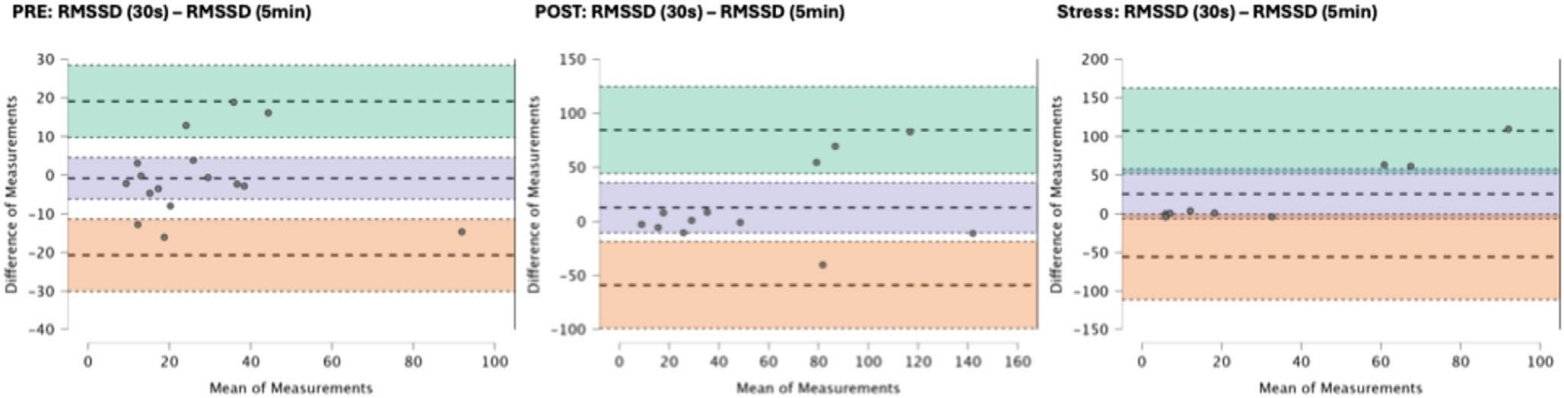
Bland–Altman plot of RMSSD by timepoint. The purple region corresponds to the mean difference and 95% CI. The green region corresponds to the mean difference +1.96SD and 95% CI. The orange region corresponds to the mean difference −1.96SD and 95% CI.

**FIGURE 5:**
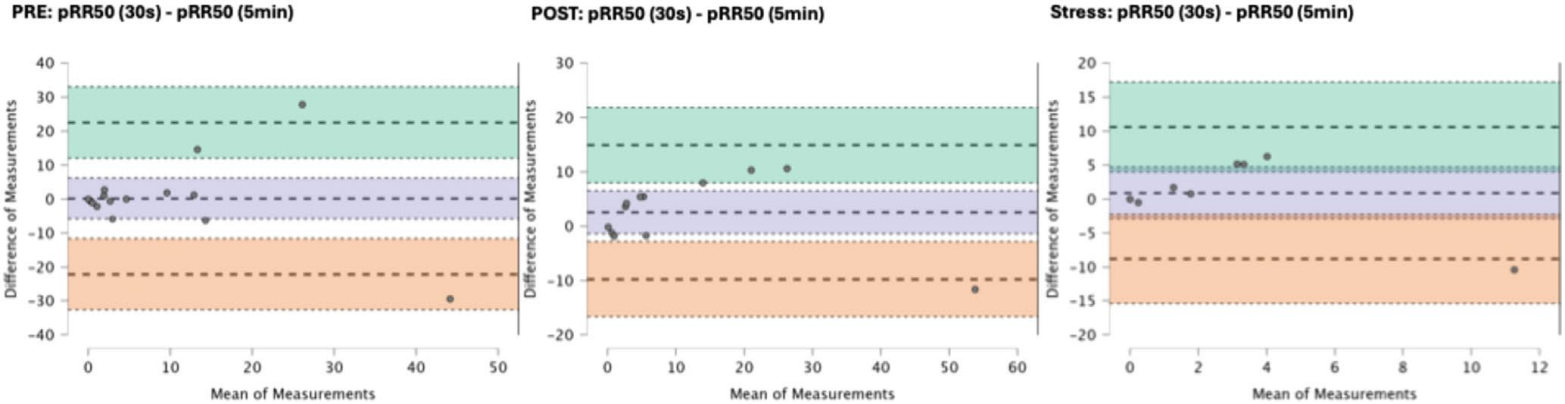
Bland–Altman plot of pRR50% by timepoint. The purple region corresponds to the mean difference and 95% CI. The green region corresponds to the mean difference +1.96SD and 95% CI. The orange region corresponds to the mean difference −1.96SD and 95% CI.

**FIGURE 6:**
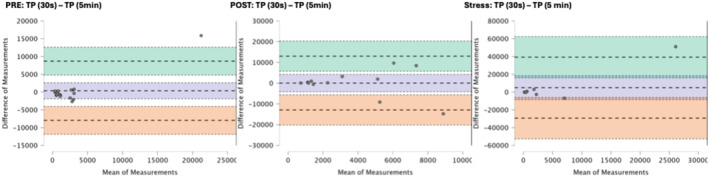
Bland–Altman plot of TP by timepoint. The purple region corresponds to the mean difference and 95% CI. The green region corresponds to the mean difference +1.96SD and 95% CI. The orange region corresponds to the mean difference −1.96SD and 95% CI.

**FIGURE 7:**
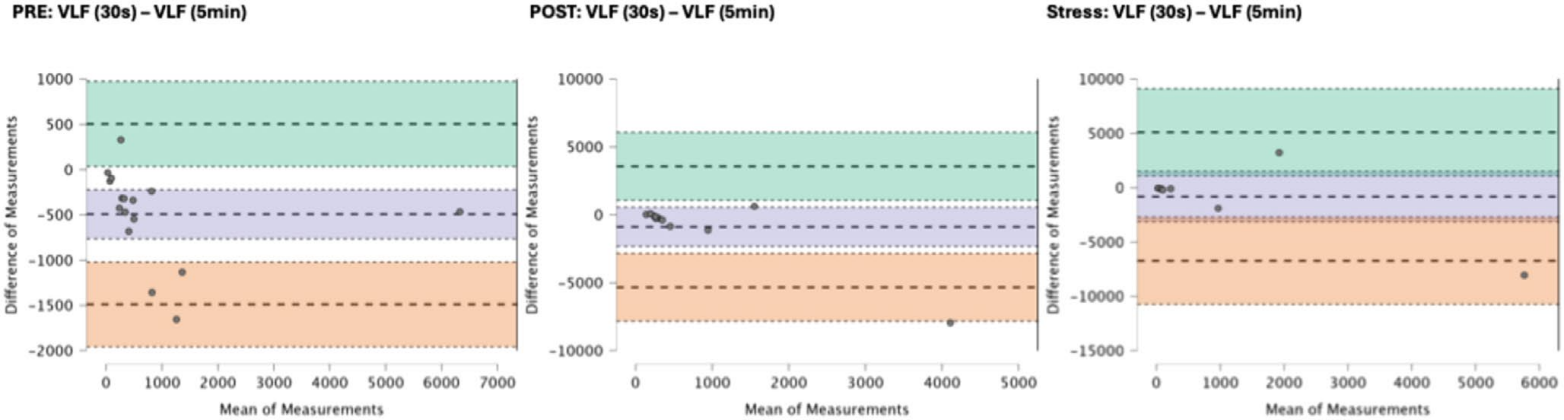
Bland–Altman plot of VLF by timepoint. The purple region corresponds to the mean difference and 95% CI. The green region corresponds to the mean difference +1.96SD and 95% CI. The orange region corresponds to the mean difference −1.96SD and 95% CI.

**FIGURE 8:**
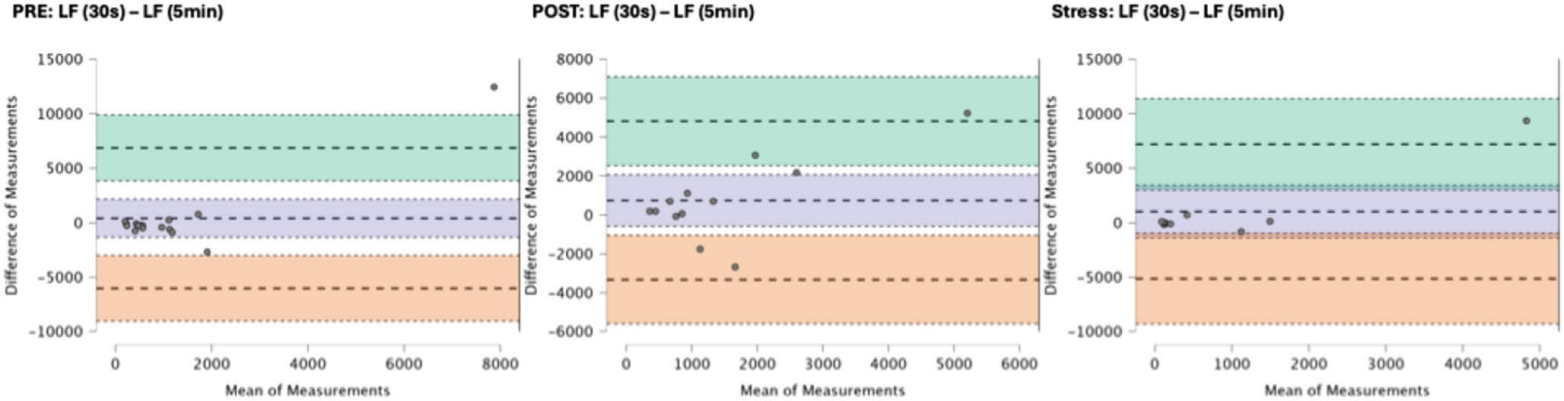
Bland–Altman plot of LF by timepoint. The purple region corresponds to the mean difference and 95% CI. The green region corresponds to the mean difference +1.96SD and 95% CI. The orange region corresponds to the mean difference −1.96SD and 95% CI.

**FIGURE 9:**
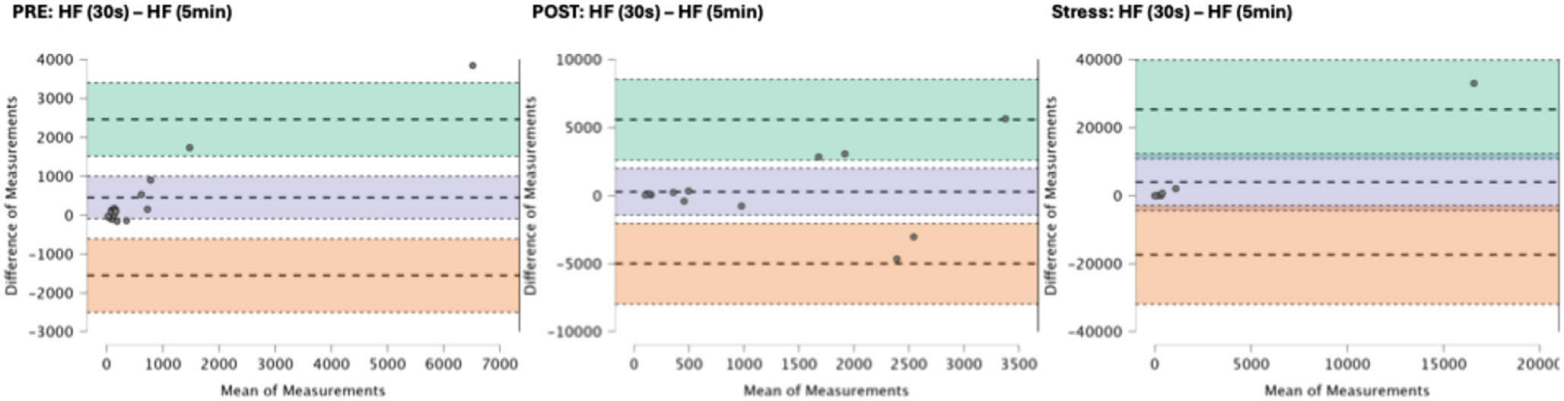
Bland Altman plot of HF by timepoint. The purple region corresponds to the mean difference and 95% CI. The green region corresponds to the mean difference +1.96SD and 95% CI. The orange region corresponds to the mean difference −1.96SD and 95% CI.

**FIGURE 10:**
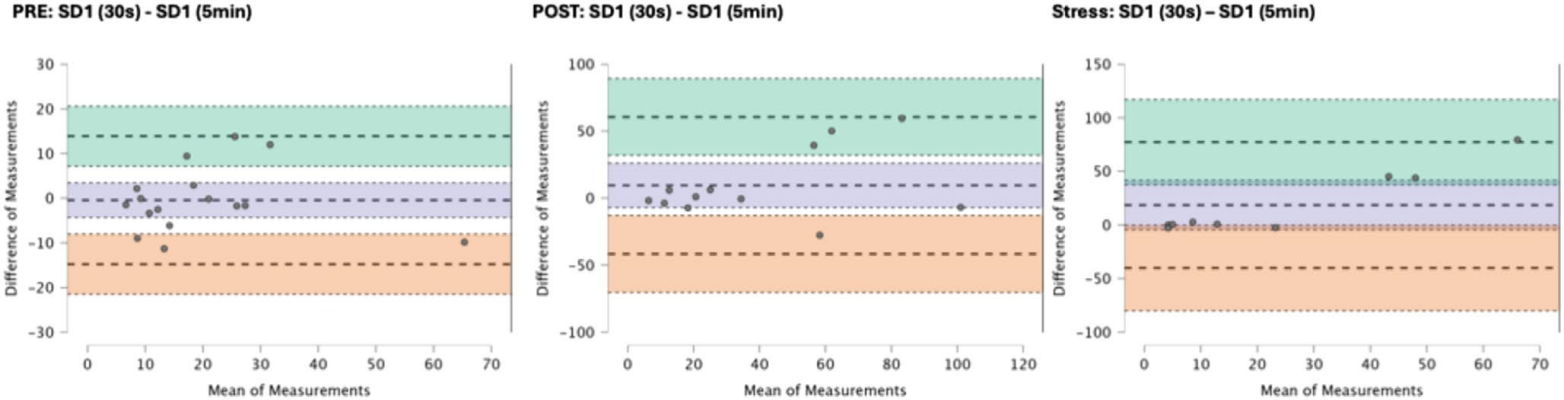
Bland–Altman plot of SD1 by timepoint. The purple region corresponds to the mean difference and 95% CI. The green region corresponds to the mean difference +1.96SD and 95% CI. The orange region corresponds to the mean difference −1.96SD and 95% CI.

**FIGURE 11:**
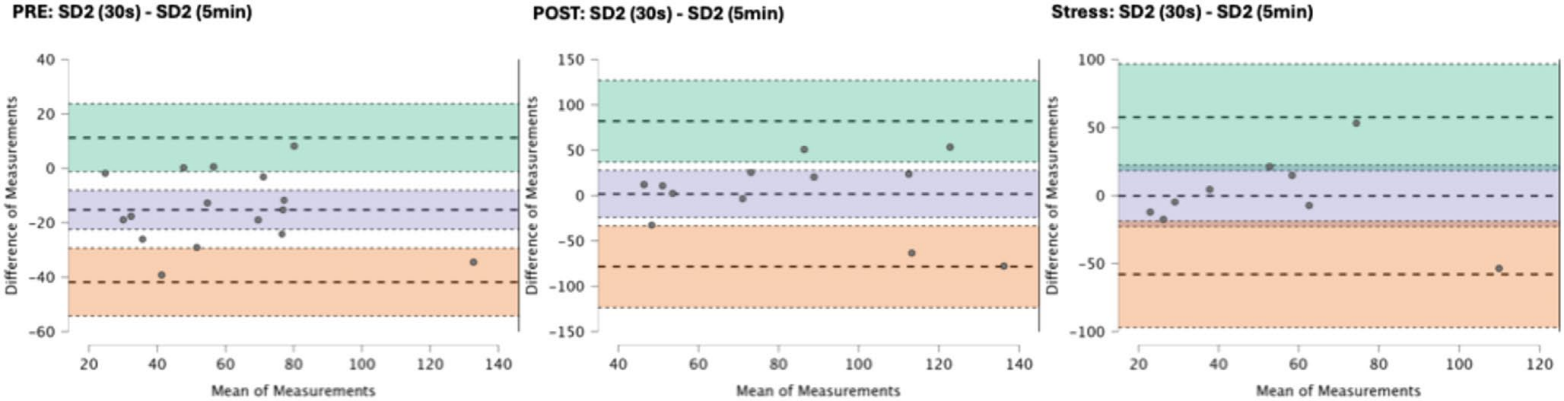
Bland–Altman plot of SD2 by timepoint. The purple region corresponds to the mean difference and 95% CI. The green region corresponds to the mean difference +1.96SD and 95% CI. The orange region corresponds to the mean difference −1.96SD and 95% CI.

**TABLE 10:** Bland–Altman Table.

HRV measure	Initial	Post‐qualification	Stress training scenario
SDRR (ms)	−10.120 (−15.440 to −4.88)	5.328 (−15.510 to 26.167)	6.733 (−16.338 to 29.805)
HR (bpm)	−2.102 (−14.208 to 10.005)	5.611 (1.881 to 9.341)	8.320 (3.189 to 13.451)
RMSSD (ms)	−0.825 (−6.234 to 4.584)	12.823 (−10.411 to 36.058)	25.748 (−6.225 to 57.721)
pRR50 (%)	0.177 (−5.895 to 6.249)	2.578 (−1.420 to 6.577)	0.898 (−2.911 to 4.706)
TP (ms^2^)	380.237 (−1882.526 to 2643.000)	69.500 (−4136.071 to 4275.071)	5006.194 (−8446.048 to 18458.437)
VLF (ms^2^)	−492.288 (−763.366 to −221.209)	−878.477 (−2323.404 to 566.449)	−809.540 (−3130.004 to 1510.924)
LF (ms^2^)	412.172 (−1340.549 to −2164.894)	743.858 (−576.185 to 2063.901)	1029.250 (1388.986 to 3447.486)
HF (ms^2^)	455.661 (−89.739 to 1001.061)	297.008 (−1417.177 to 2011.192)	3995.020 (−4382.328 to 12372.368)
SD1 (ms)	−0.453 (−4.346 to 3.441)	9.526 (−7.054 to 26.106)	18.554 (−4.475 to 41.584)
SD2 (ms)	−15.253 (−22.468 to −8.038)	1.838 (−24.166 to 27.842)	−0.163 (−22.787 to 22.460)

*Note*: All values are reported as mean measurement differences (95% CI).

Abbreviations: HF, High frequency; HR, Heart rate; LF, Low frequency; RMSSD, Root‐mean square of successive differences; SD1, Poincare plot standard deviation along the line perpendicular to the identity; SD2, Poincare plot standard deviation along the line of identity. SDRR, Standard deviation of R‐R Intervals; TP, Total Power; VLF, Very Low frequency.

## 4 DISCUSSION

The primary aim of this study was to assess the reliability of 30‐s versus 5‐min HRV measurements in a cohort of SWAT personnel throughout a day of qualification and training events. While police personnel are at a potentially high risk of allostatic load due to the high‐stress nature of their work environments, human performance optimization efforts that may begin with assessment, such as HRV monitoring, remain difficult to incorporate in context. This may be particularly true if ECG systems are not wireless or are to be incorporated in active stations where changes in events are less predictable than in training context. Therefore, reducing the logistical burden of these efforts by reducing the time requirement to obtain valuable health and performance data may benefit personnel, organizations, and communities, but expedience should not supplant fidelity of the data obtained. Indeed, the chief consideration of shortening timeframes for HRV analysis is the potential for insufficient description of longer‐term, such as a circadian influence, fluctuations (Gronwald et al., 2024). Previous research suggests that 24‐h HRV analyses are not interchangeable with 5‐min (short‐term) HRV assessments. This study sought to assess the reliability and agreement between short‐term (ST, 5‐min) and ultra‐short‐term (UST, 30‐s) HRV analyses.

Based on the results of the ICC assessments in this study, the 30‐s window is only reasonably reliable (as per the Koo & Li criteria) compared to the 5‐min ECG recording when assessing HRV in conditions of relative rest, and metrics should be chosen carefully. In this study, as the participants progressed throughout the day of qualification and training, fewer HRV metrics demonstrated acceptable reliability (ICC>0.75). Conversely, HR, while not reliable at baseline, proved to be highly reliable as the day progressed. This reversal in reliability of HR and HRV may be explainable by the stability of HR in response to the increase in loads throughout the day (Brockmann & Hunt, 2023). Indeed, HR did appear to increase at each progression from qualification to stress event exposure. While not adequately assessed in this study, it is reasonable that circadian changes may also have influenced changes in reliability. The metrics that did demonstrate acceptable reliability at baseline included SDRR, RMSSD, VLF, HF, SD1, and SD2. Only pRR50% was reliable post‐qualification, and no measures of HRV were reliable following the stress event. Conversely, the Bland–Altman plots indicated inadequate reliability across all measures. Granted, the presence of outliers primarily drives this conclusion, but at least two cases lie outside the 95% CI of the mean in all instances.

The time domain measures relevant in this study determine HRV in a similar manner, in that both calculate variation in RRIs over time. The SDRR metric assesses standard deviation of all RRIs in the epoch, whereas RMSSD calculates variability in adjacent beats. Greater RMSSD and SDRR both generally indicate greater cardiovascular health and fitness, but SDRR may be more sensitive to longer‐term fluctuations (Shaffer & Ginsberg, 2017). For the frequency domain measures, which assess HRV by decomposing variability into constituent frequencies, the purpose of utilizing the HF (high frequency) domain is to assess parasympathetic activity and cognitive regulatory states dependent on resource availability and environmental demands (Stephenson et al., 2021). It is important to note, though, that the HF domain is not exclusively composed of parasympathetic activity (Billman, 2013), and while decreasing HF values are known to be associated with overstress (Tomes et al., 2020), it does not purely indicate vagal withdrawal. In the context of sports physiology and high‐intensity activities, SD1 provides insights into short‐term HRV when signals contain noise (Shaffer & Ginsberg, 2017) and is useful as a surrogate for other short‐term measures. Like other short‐term measures, SD1 aims to reflect the influence of the parasympathetic nervous system on heart rate regulation during demanding physical tasks (Stephenson et al., 2021). Monitoring SD1 in physically demanding settings may improve assessment of dynamic changes in autonomic nervous system activity. Again, it should be noted that while vagal withdrawal may be detectable with the SD1 measure, it is not entirely comprised of parasympathetic activity only (Billman, 2013).

While 30‐s long‐term measures were reliable as compared to the 5‐min measure for VLF as assessed by ICC, this finding may be erroneous, as the 5‐min measure itself may not have been sufficient to accurately characterize these components of HRV (Sammito et al., 2015). This finding is supported by the results of the Bland–Altman plot, which indicated very poor reliability for the VLF measure, particularly at baseline. Specifically, VLF aims to characterize long‐term fluctuations in cardioregulatory activity, such thermoregulation, fluctuations in the activity of the renin‐angiotensin‐aldosterone system, and peripheral chemoreceptor responses (Usui & Nishida, 2017; Weber et al., 2010). SD2 may also have been erroneously reliable in this study, although SD2 does capture both short‐term and long‐term oscillatory patterns.

In summary, these findings generally agree with previous research in that the SD1 domain is highly robust (Tomes et al., 2020). Indeed, nonlinear HRV appears to be the most suitable measure in contexts where noise or interruptions in data collection are likely (Shaffer & Ginsberg, 2017), and as the overall quantity of available data are reduced, measures such as SD1 maintain a greater degree of fidelity than time‐domain or frequency‐domain measures. This study does have its limitations, however, in that the sample size was small by conventional standards. It should be noted, though, that in research with SWAT personnel, the number is not unreasonable. Further, the smaller sample size may actually be beneficial for the intent of this study, as the objective of this research was to consider the reliability of HRV assessment with varying lengths of input data. Therefore, in smaller organizations, these findings are of greater translational value. Additional limitations include the lack of controlling for certain confounders, such as sleep duration and caffeine, which are known to influence HRV. However, this does represent real‐world pragmatic application as would be the case if used in these populations. In regards to sleep specifically, some of the operators (*n* = 10) were on‐duty the night before the study, meaning they were conducting police work overnight outside of the SWAT role. For those operators not on duty (*n* = 6), sleep was permitted, but not measured. Regardless though, with the objective of this study to determine reliability of 30‐s versus 5‐min ECGs from a single overall ECG, for the 30‐s window to be preferable, it should be robust to such confounders.

## 5 CONCLUSIONS

The findings of this study indicate that in general, HRV should ideally be measured from ECG data of at least 5 min in length. However, for some time domain, nonlinear and HF spectral analyses, 30 s may be sufficiently reliable provided individuals are relatively at rest. This research offers a starting point for organizations interested in implementing data‐informed health and human performance efforts, but may have limited time available to dedicate to such measures. Further studies may consider the minimum acceptable measurement time for long‐term HRV in this setting, as the reliability of those indices (VLF and SD2) were likely both unreliable.

## AUTHOR CONTRIBUTIONS

Conceptualization, C.D.T., E.F.D.C., and R.O.; methodology, E.F.D.C. and C.D.T.; software, E.F.D.C. and C.D.T.; validation, R.O. and B.S.; formal analysis, C.D.T.; investigation, E.F.D.C. and C.D.T.; resources, C.D.T., R.O., and B.S.; data curation, R.O. and C.D.T.; writing—original draft preparation, C.D.T.; writing—review and editing, E.F.D.C., R.O., and B.S.; visualization, C.D.T.; supervision, R.O., B.S., and E.F.D.C.; project administration, R.O. and B.S. All authors have read and agreed to the published version of the manuscript.

## FUNDING INFORMATION

This research was supported by a PhD scholarship awarded to the lead author by Bond University (award number 13461098). No other funding or grant from any agency in the public, commercial or not‐for‐profit sectors was provided or otherwise obtained.

## CONFLICT OF INTEREST STATEMENT

The authors declare no conflicts of interest. The funders had no role in the design of the study; in the collection, analyses, or interpretation of data; in the writing of the manuscript; or in the decision to publish the results.

## ETHICS STATEMENT

The study was conducted in accordance with the Declaration of Helsinki and approved by the Bond University Human Research Ethics Committee (BUHREC) (Protocol 2019–022 amnd 2).

## INFORMED CONSENT STATEMENT

Informed consent was obtained from all subjects involved in the study.

## Data Availability

The datasets supporting the conclusions of this article are not publicly available as data were obtained from a law enforcement agency, and as per research ethics provisions, individual participant data cannot be released without a specific request to, and approval from, the sponsoring agency. To make a request, or for further information, please contact Dr. Rob Orr, Bond University Tactical Research Unit; rorr@bond.edu.au.
